# Molecular epidemiology of *Klebsiella variicola* obtained from different sources

**DOI:** 10.1038/s41598-019-46998-9

**Published:** 2019-07-23

**Authors:** Humberto Barrios-Camacho, Alejandro Aguilar-Vera, Marilu Beltran-Rojel, Edgar Aguilar-Vera, Josefina Duran-Bedolla, Nadia Rodriguez-Medina, Luis Lozano-Aguirre, Olga Maria Perez-Carrascal, Jesús Rojas, Ulises Garza-Ramos

**Affiliations:** 1 Instituto Nacional de Salud Pública (INSP), Centro de Investigación Sobre Enfermedades Infecciosas (CISEI), Laboratorio de Resistencia Bacteriana, Cuernavaca, Morelos, Mexico; 2 Universidad Nacional Autónoma de México, Centro de Ciencias Genómicas, Programa de Genómica Funcional de Procariotes, Cuernavaca, Morelos, Mexico; 3 Instituto Nacional de Salud Pública (INSP), Centro de Información para Decisiones en Salud Pública (CENIDSP), Cuernavaca, Morelos, Mexico; 4 Universidad Nacional Autónoma de México, Centro de Ciencias Genómicas, Programa de Genómica Evolutiva, Cuernavaca, Morelos, Mexico

**Keywords:** Microbiology, Molecular biology, Bacteria, Bacterial genomics

## Abstract

*Klebsiella variicola* is considered an emerging pathogen in humans and has been described in different environments. *K. variicola* belongs to *Klebsiella pneumoniae* complex, which has expanded the taxonomic classification and hindered epidemiological and evolutionary studies. The present work describes the molecular epidemiology of *K. variicola* based on MultiLocus Sequence Typing (MLST) developed for this purpose. In total, 226 genomes obtained from public data bases and 28 isolates were evaluated, which were mainly obtained from humans, followed by plants, various animals, the environment and insects. A total 166 distinct sequence types (STs) were identified, with 39 STs comprising at least two isolates. The molecular epidemiology of *K. variicola* showed a global distribution for some STs was observed, and in some cases, isolates obtained from different sources belong to the same ST. Several examples of isolates corresponding to kingdom-crossing bacteria from plants to humans were identified, establishing this as a possible route of transmission. goeBURST analysis identified Clonal Complex 1 (CC1) as the clone with the greatest distribution. Whole-genome sequencing of *K. variicola* isolates revealed extended-spectrum β-lactamase- and carbapenemase-producing strains with an increase in pathogenicity. MLST of *K. variicola* is a strong molecular epidemiological tool that allows following the evolution of this bacterial species obtained from different environments.

## Introduction

The *Klebsiella* genus, a member of the family *Enterobacteriaceae*, comprises species found in diverse environmental niches. In fact, using phylogenetic reconstruction methods, *Klebsiella pneumoniae* has been divided into five distinct species. *Klebsiella variicola* was first described in 2004^1^, followed by the identification *Klebsiella quasipneumoniae* in 2014 (with two subspecies; *K. quasipneumoniae* subsp. *quasipneumoniae* and *K. quasipneumoniae* subsp. *similipneumoniae*)^2^, *Klebsiella quasivariicola* (which remains to be validated) in 2017^3^. Finally in 2019 *Klebsiella africanensis* a new bacterial species and a subspecies of *K. variicola*; named, *Klebsiella variicola* subsp. *tropicalensis* were described^4^. The description of these new bacterial species has expanded the taxonomic classification of the genus *Klebsiella*, which are described as the *Klebsiella pneumoniae* complex^5^. Since *K. variicola* was described several international reports have discussed its importance^6^; indeed, it is considered an emerging pathogen in humans^7^. Similar to other *Klebsiella* species, *K. variicola* is a gram-negative, facultative anaerobic, nonspore-forming, nonmotile rod-shaped bacteria that forms circular, convex, and smooth colonies^8^. *K. variicola* was initially identified as an endophyte in plants and as a pathogen in humans^1^. In addition, *K. variicola* is considered a symbiont in insects, a pathogen in animals and plants. Moreover, *K. variicola* has been identified in several environmental sources^6,9–11^. As a human pathogen, *K. variicola* has been isolated from diverse clinical samples, including the blood, tracheal aspirates, several types of secretions, the respiratory and urinary tract infections, and surgical wounds^7^. The estimated prevalence of *K. variicola* is highly variable: initially, a prevalence of 8% was reported^1^, which has varied over time from 1.8% to 24.4% in clinical settings^12–14^. The highest percentage reported to date is 24.4%, which were obtained from bloodstream infections in a University Hospital in Solna, Sweden^14^. The prevalence of the species complex is variable, mainly due to misclassification problems^13^.

Members of the *K. pneumoniae* complex share biochemical and phenotypic features. This has led to misclassification by conventional methods and several cases of *K*. *variicola* misidentified as *K. pneumoniae* and in a few cases as *K. quasipneumoniae; K. quasipneumoniae* has also been misidentified as *K. variicola*^15,16^. *K. pneumoniae* being the most prevalent species within the complex^4,13,17^, however, regarding urinary tract infections, *K. variicola* has been isolated more frequently unlike *K. pnuemoniae* and *K. quasipneumoniae*^18^.

Phylogenetic analysis of the *Klebsiella* genus, the *rpoB* gene has been recommended for the proper differentiation of this genus^19^, even though both the 16S rRNA and *rpoB* genes have been used for this purpose^14,20^. The *K. variicola* strain DX120E was identified using these genes, with *rpoB* showing a higher level of accuracy^21^. As correct identification of *K. variicola* using phylogenetic analysis requires time and trained personnel, thus, several biochemical and basic molecular methods have been explored. The biochemical method using adonitol was not effective, generating false positives^22^. Nonetheless, several PCR methods have been developed to differentiate certain species of the *Klebsiella* genus^12,23–25^. In addition, the use of Matrix-Assisted Laser Desorption/Ionization-Time of Flight (MALDI-TOF) mass spectrometry to identify microorganisms has frequently been reported^26^. Despite initial difficulty in differentiating among members of this genus^13,23^, the MALDI-TOF approach has been recently optimized, particularly for the *K. pneumoniae* complex^5^.

Using PCR screening, phylogenetic analyses and whole-genome sequencing (WGS) methods, *K. variicola* has recently been identified in diverse niches with clinical and environmental importance^6,7,15^. These efforts to identify and characterize a significant number of *K. variicola* isolates have prompted studies of their molecular characterization and epidemiology. The present study describes the molecular epidemiology of *K. variicola* using Multilocus Sequence Typing (MLST), developed for this purpose. This study identified broad dissemination of *K. variicola* isolates obtained from different regions of the world and a considerable number of ESBL- and carbapenemase-producing isolates were identified. Likewise, a possible pandemic clone was identified and the notion of kingdom-crossing bacteria from plants to humans, establishing this as a route of transmission for *K. variicola*.

## Results and Discussion

The *K. variicola* genomes were acquired from public databases, which were collected from various sources in several countries of the five continents. The isolates include from plants, insects, the environment, animals, and a significant number of isolates were obtained from human samples (Supplementary Dataset). Based on 33 *K. variicola* genomes, the AMPHORA program identified a set of 31 phylogenetic marker genes. Among these genes, *rpoB, phoE, nifH, mdh*, and *infB* were previously considered for phylogenetic analysis in *K. variicola*^1^, and six genes (*phoE*, *tonB*, *rpoB, mdh, infB*, and *gapA*) are included in the *K. pneumoniae* MLST^27^. In addition, concatenation of *fusA*, *gapA*, *gyrA*, *leuS* and *rpoB* has been proposed for the proper phylogenetic differentiation of *K. pneumoniae*, *K. variicola* and *K. quasipneumoniae*^2^. The genes *gyrA*, *nifH* and *tonB* were eliminate due to they may be subjected to selection bias either by the use of antimicrobial agents^28^, nitrogen fixation^1,29,30^ or binding and transport of ferric chelates^31^, respectively. Finally, *leuS* (leucyl-tRNA synthetase), *pgi (*phosphoglucose isomerase), *pgk (*phosphoglycerate kinase), *phoE* (phosphoporine E), *pyrG* (CTP synthase), *rpoB* (β-subunit of RNA polymerase B) and *fusA* (elongation factor G) were selected for *K. variicola* MLST scheme (Table 1) (http://mlstkv.insp.mx) and for the assignation of sequence types (ST) (Supplementary Dataset). Of note, the *pyrG* (CTP synthase class I) gene is not present in *K. pneumoniae* genomes, which was verified using a BLASTn genome search and confirmed by PCR of several *K. pneumoniae* clinical isolates in our collection (data not shown). The primer sequences, amplified fragments, number of alleles, nucleotide diversity and polymorphic sites of each of the seven genes are described in Table 1.

**Table 1:** Characteristics of genes, primers, PCR-conditions, nucleotide diversity and polymorphic sites for the *K. variicola* MLST scheme.

Locus	Function	Primer name	Primer sequence	Temp (°C)	Size (bp)	No. of alleles	Nucleotide diversity	Polymorphic sites (nonsynonymous substitutions)
*leuS*	Leucyl-tRNA synthetase	leuSKv-F	CGAACAGGTTATCGACGGCT	63	594	48	0.012462	48 (8)
		leuSKv-R	CAAAGGTGTCGGTTTCACGC					
*pgi*	Phosphoglucose isomerase	pgiKv-F	AAAGAGACCGATCTGGCAGG	60	600	42	0.016425	49 (6)
		pgiKv-R	ACCAGATACCGATCAGCGCC					
*pgk*	Phosphoglycerate kinase	pgkKv-F	TCGTGATGGATGCTTTCGGT	63	444	26	0.012647	33 (10)
		pgkKv-R	AGATTTTGTCAGCGATGCCG					
*phoE*	Phosphoporine E	phoEKv-F	CTGTACGACGTGGAAGCCTG	63	453	54	0.018569	46 (10)
		phoEKv-R	CCACGAAGGCGTTCATGTTT					
*pyrG*	CTP synthase	pyrGKv-F	CCGATCGCTATGGTCGCTG	60	522	60	0.011535	48 (22)
		pyrGKv-R	CGGGACATCAGTTCCGGGT					
*rpoB*	β-subunit of RNA polymerase B	rpoBKv-F	GCCAGCTGTCCCAGTTTATG	60	513	25	0.014945	38 (3)
		rpoBKv-R	GAACGGTACCGCCACGTTTA					
*fusA*	Elongation factor G	fusAKv-F	CGAAAACCAAAGCTGACCAGG	62	561	15	0.007130	23 (4)
		fusAKv-R	CATGGTGTATGATGCACGACCT					

**Figure 1:**
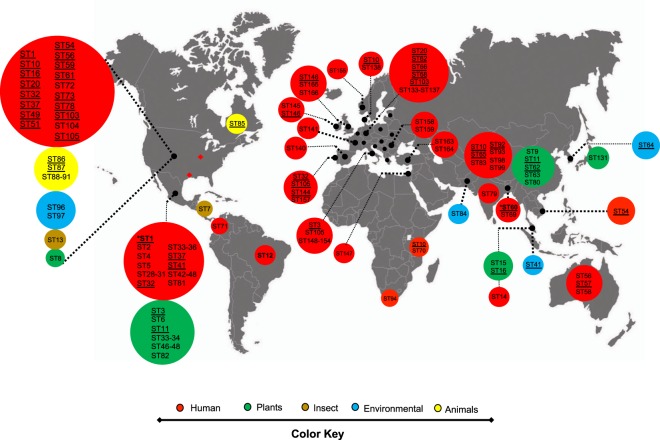
Molecular epidemiology of *K. variicola* isolates. The map shows the localization of each ST. The underlined ST corresponds to ST with two or more isolates. The major ST corresponds to genomes described in the USA, Mexico, Germany and China. The origin of the isolates is shown in color codes. The ST market with asterisks corresponds to *K. variicola* outbreaks. The WGS projects of *Klebsiella* in the USA are marked with a diamond, corresponding to Texas and Missouri. The black circles in Europe correspond to countries described with a single or two ST (ST underline), as: Greece (ST86), Austria ST125, Belgium (ST132), Estonia (ST139), Croatia (ST142 and ST143), Hungary (ST144), Poland (ST156), Serbia (ST160) and Slovenia (ST161 and ST162).

Considering *K. variicola* isolate 801 obtained from a pediatric outbreak in Mexico^32^ as ST1 (Supplementary Dataset), and the MLST was applied arbitrarily to *K. variicola* genomes and isolates include in the study. A total of 166 distinct sequence types (STs) were identified among 254 *K. variicola* genomes and isolates obtained from different sources, such as humans, plants, insects, the environment and animals. From 166 STs, 39 STs were shared by at least two isolates (Fig. 1 and Supplementary Dataset).

The global distribution of STs is shown in Fig. 1. The major number of isolates assigned to an ST were from the USA, followed by Mexico, China and Europe (mainly Germany). In the case of the USA, numerous isolates with the same ST were also identified in other regions of the world (Supplementary Dataset). In particular, *Klebsiella* WGS projects have been performed in the USA, the first was carried out by the Houston Methodist Hospital, Texas^13^ and the second from the Barnes-Jewish Hospital microbiology laboratory in Missouri^18^. In both cases, numerous *K. variicola* isolates were identified, with ST49, ST51, ST73, ST75 and ST77 described in both works (Fig. 1 and Supplementary Dataset).

With respect to Mexico, ST1 corresponds to the first pediatric outbreak of *K. variicola*^32^ and other isolates obtained from the USA (WUSM_KV_09)^18^. In addition, this country contributed the most isolates obtained from different plants (Fig. 1). Overall, human isolates are heterogeneous regarding STs, and only ST32 was identified for two human isolates. Nevertheless, the ST37 and ST41 contains isolates from Mexico, USA and Singapore. In China, isolates from both humans and plants have been described, with ST65 and ST92 corresponding to human isolates described in different reports (Fig. 1 and Supplementary Dataset). The BioProject-PRJEB10018 includes *Klebsiella* isolates from European countries and identified numerous *K. variicola* human isolates. These isolates showed heterogeneous STs, with only ST144 (Hungary and Portugal) and ST146 (Ireland and United Kingdom) having the same ST. However, countries in different regions of the world harbor the same STs (ST3, -20, -32, -37, -68, -77, -86, -105 and -125) of isolates described in Europe.

In particular, some STs are highlighted, such as ST10 identified in Denmark, China, Tanzania and the USA. ST11 isolates obtained from plants originate from Mexico and China. Nine human isolates obtained from Germany, Belgium and the USA were identified as ST20^18^, and ST56 and ST57 were found on distant continents such as North America, Europe and Australia, all from human samples. ST60 corresponds to the second pediatric outbreak of *K. variicola* described in Bangladesh^33^ and ST64 to *K. variicola* obtained from the environment in South Korea^10^.

Interestingly, three cases of kingdom-crossing bacteria (KCB) were identified by MLST. ST3 was identified in a plant (banana roots) isolate in Mexico and human isolates from Italy and Serbia. ST16 was identified in isolates obtained from a chili plant in Malaysia and humans in the USA. ST62 corresponds to a well-characterized *K. variicola* D5A isolate obtained from plants in China^34^ and ID_24 isolates obtained from humans in Germany. The proposal of KCB from plants to humans, which may indicate a process of transfer known as phytonosis, has been described previously^29^. *K. variicola* clinical isolate X39 is considered a KCB because it contains genes involved in plant colonization, nitrogen fixation, and defense against oxidative stress; this isolate is also considered an endophytic bacterium based on its capacity to colonize maize^35^.

**Figure 2:**
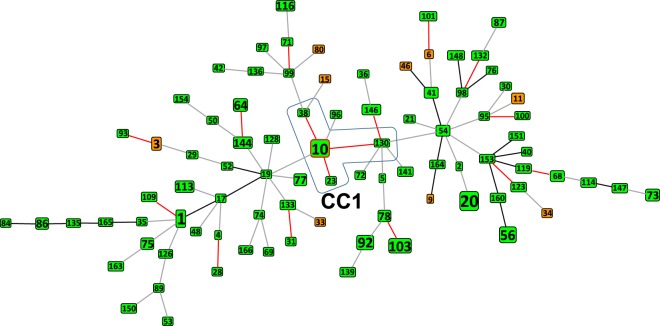
goeBURST analysis of *K. variicola* isolates. Single-locus variants (SLVs) are in red, double-locus variants (DLVs) are in black, and triple-locus variants (TLVs) are in grey. The founder ST10 and ST38, ST23 and ST130 with an SLV correspond to Clonal Complex 1 (CC1). The isolates from humans and plants are in green and orange squares, respectively. The size of a node is proportional to the number of isolates presenting that ST in the database (Supplementary Dataset).

*K. variicola* MLST allowed us to analyze the allelic profile of the *K. variicola* isolates included in this study using the goeBURST algorithm. Figure 2 shows the sequence types of major isolates and ST relatedness. A total of 166 STs were identified from among the 254 isolates included in this study, and 127 are unique. The founder ST10 and ST23, ST38 and ST130 with a single locus variants (SLVs) comprise Clonal Complex 1 (CC1). ST10 corresponds to one of the most predominant STs, representing 70% of all strains in CC1 (7/10). Moreover, all CC1 strains have a human origin, and they were obtained from five different countries (China, Denmark, Mexico, Tanzania and the USA). In addition to CC1, other 12 SLVs (42 isolates) were identified by goeBURST analysis and only three SLV were from the same country. Approximately 85% of the SLVs were isolated from humans, 9% from the environment and 5% from plants. None of these SLVs from plants or the environment share a relationship. All STs described above present an SLV relationship, without sharing a clear common origin or country. The same heterogeneity was observed for 17 double locus variants (DLVs) (40 isolates) (Fig. 2).

**Figure 3:**
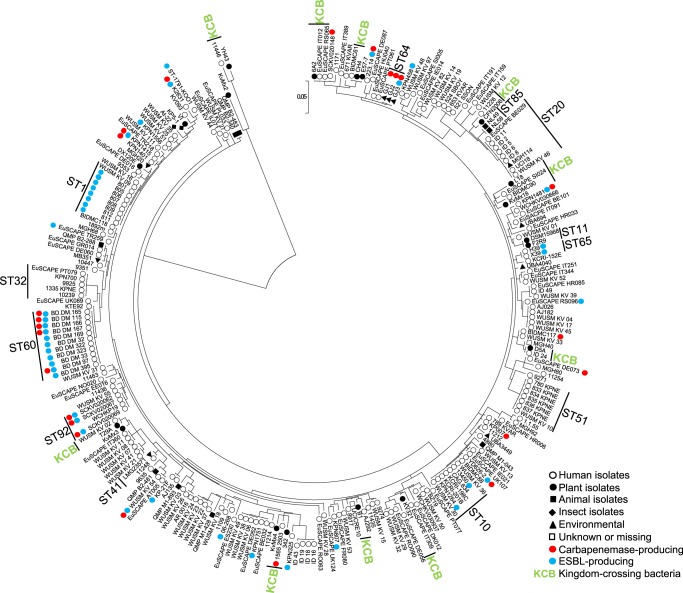
Phylogenetic tree of *K. variicola* isolates obtained from different sources. The tree includes the seven concatenated genes from genomes and the isolates described in the present study. Isolates associated with human infection are indicated by white circles, endophyte and rhizosphere isolates with black circles, isolates obtained from animals with black square, insect isolates with black diamond, environmental isolates with black triangles and isolates with unknown or missing origin with white squares. ESBL- and carbapenemase-producing isolates are represented by blue and red circles, respectively. KCB corresponds to kingdom-crossing bacteria identified in the analysis.

*K. variicola* isolates obtained from different sources are suggested to be derived from a common genetic pool, without segregation between isolates from these sources^13,36^. In addition, Potter *et al*.^18^ reveal two distant lineages among *K. variicola* genomes using phylogenetic analysis of the core genome. In this study, the phylogenetic analysis was carried out using the seven concatenated genes from *K. variicola* MLST (Fig. 3). Similarity to previous works, showed not segregate isolates with regard to origin of the sample and the distant lineage was formed by the same *K. variicola* KvMx2 and YH43 isolates obtained from sugarcane and potato plants, respectively. In addition, in this distant lineage also grouped the *K. variicola* 11446 isolate obtained from humans (Fig. 3). The YH43 isolate is another case of misclassification of *K. variicola*, which was described as *K. pneumoniae*^37^.

The isolates obtained from public data bases were mostly obtained from humans (84.6%), followed by plants (7.0%), animals (3.5%), the environment (3.1%), insects (0.7%) and unknown or missing origin (0.7%). Clusters that corresponds to isolates of the same ST and KCB were revealed by phylogenetic analysis (only the most representatives are indicated in the Fig. 3). Isolates from human and plants phylogenetically related and that may be correspond KCBs are showed; these isolates are as follows: 11226 and CFN2006; L18, EuSCAPE_SI024, BIDMC90 and KvMx18; EuSCAPE_IT309 and KV321; AJ292 and B1; 342, 1565/2503 and KvMx4; WUSM_KV_02 and T29A and YH43 and 11446. Moreover, the At-22^31^ and KP5-1^38^ isolates obtained from insects, together with the VI isolates from plants^1^, are phylogenetically related. However, MB351 obtained from the environment (industrial effluent), EuSCAPE_DE060 and EuSCAPE_GR014 from humans and QMP_B2_288 from an animal (bovine) are phylogenetic related. Interestingly, isolates 11248 and LMG23571 obtained from humans and the environment in Mexico and Singapore, respectively, belong to ST41 (Supplementary Dataset and Fig. 1).

**Figure 4:**
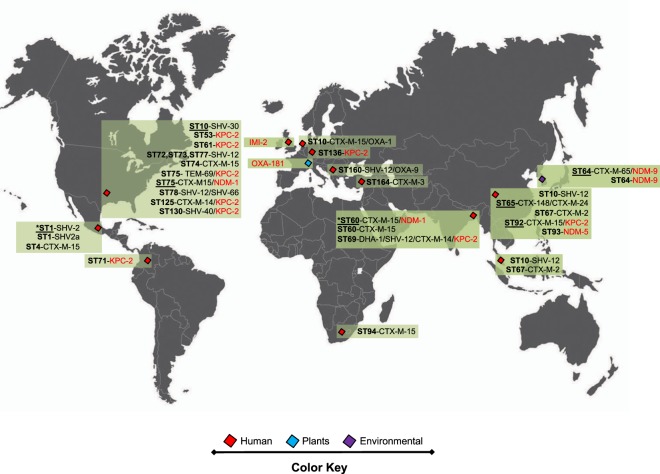
Molecular epidemiology of ESBL- and carbapenemase-producing *K. variicola* isolates. Underlined STs contain several isolates. ST and ESBL- and/or carbapenemase-producing genes corresponding to *K. variicola* outbreaks are marked with asterisks. The origin of the isolates is shown in color codes. The IMI-2 and OXA-181 carbapenemase-producing *K. variicola* isolates lack WGS data.

The molecular epidemiology of ESBL- and carbapenemase-producing *K. variicola* isolates were explored using published data and for unpublished genomes the acquisition of resistance to β-lactam antibiotics due to β-lactamases was determined *in silico* (Fig. 4) (see Material and Methods). ESBL-producing isolates belong to ST1 (8/9 isolates), ST4, ST10 (2/7 isolates), ST14, ST57 (1/2 isolates), ST60 (6/11), ST64 (1/3 isolates), ST65 (2/2 isolates), ST69, ST72, ST74, ST76, ST77 (1/2 isolates), ST78 (1/2 isolates), ST92 (3/4 isolates), ST94, ST125 (1/2 isolates), ST130, ST160 and ST164. ESBLs SHV-type and CTX-15 were the most prevalent (Fig. 4 and Supplementary Dataset). Several ST described above also are carbapenemase-producing isolates, which have been described in different countries. In the USA, ST53, ST61, ST75, ST125 and ST130 were found to be KPC-2 producers and, in some cases, in combination with ESBL SHV- or CTX-M-type strains. Similarly, ST76 produces NDM-1 and CTX-M-15. Another carbapenemase-producing *K. variicola* isolates on the American continent are ST71 with KPC-2. In Europe, ST136 with KPC-2 has been reported. In Asian countries, ST60 corresponds to the pediatric outbreak in Bangladesh and is composed of CTX-M-15 and NDM-1 producers and the ST69 in this country produces ESBLs and KPC-2. In South Korea, ST64 obtained from river water was positive for NDM-9 and CTX-M-65 in some isolates. Regarding China, ST92 and ST93 produce KPC-2 and NDM-5, in combination with CTX-M-15 for ST93. Half of the isolates described as carbapenemase-producing also were positive for ESBLs of TEM-, SHV- or CTX-M-type families (Fig. 1 and Supplementary Dataset).

Although no WGS data are available in two reports of carbapenemase-producing *K. variicola* isolates^39,40^, we would like to highlight these studies because they correspond to the first descriptions of carbapenemase-producing *K. variicola* isolates. The plasmid-borne carbapenemase genes identified were OXA-181 and IMI-2, from Switzerland and the United Kingdom, respectively.

Currently, accurate differentiation of *K. pneumoniae*, *K. quasipneumoniae*, *K. variicola* and *K. quasivariicola* is not routinely performed in the clinical setting. The main reason is the lack of implementation of the methods available in clinical or research laboratories^41^. Therefore, any of the *K. quasipneumoniae*, *K. variicola* and *K. quasivariicola* isolates are continuously misclassified as *K. pneumoniae*. The clinical importance of *K. variicola* is overshadowed by inaccurate identification, and thus, the actual prevalence has also been underestimated. Initially, correctly identified *K. variicola* was achieved by phylogenetic analysis mainly using the *rpoB* gene. However, several molecular approaches have been proposed to properly detect these species^12,23,24^, and MALDI-TOF identification of these species has been improved^5^. In addition, one-step PCR amplification of chromosomal β-lactamases of *K. pneumoniae*, *K. quasipneumoniae* and *K. variicola* for identification should be considered carefully. Long *et al*.^13^ identified a rare recombinant of the OKP/LEN core genes. In the present work, the LEN-type gene was found in 98.8% of the *K. variicola* genomes, whereas the other 1.2% of the genomes lacked for LEN-type chromosomal β-lactamase (Supplementary Dataset), a fact that should be considered when chromosomal β-lactamase genes are used for species identification. Nevertheless, at the genomic level, misclassifications among *K. pneumoniae*, *K. quasipneumoniae* and *K. variicola* have recently been rectified^15^. An excellent option at the genomic level is ANI, a tool proposed for the correct identification of bacterial species^15,42,43^. Overall, the increasing number of options for differentiating *K. variicola* from closely related species in the *K. pneumoniae* complex increases the number of isolates, and more WGS projects for this bacterial species will likely be conducted in the near future.

Considering the findings described above, the ANI tool was implemented in our MLST scheme for *K. variicola* (http://mlstkv.insp.mx) for correct identification of bacterial species using WGS data. These WGS data is compared with the reference genomes of *K. pneumoniae*, *K. quasipneumoniae*, *K. variicola* and *K. quasivariicola*, and once ANI confirme that the genome correspond to *K. variicola* (>95%), the ST is assigned according to the *K. variicola* MLST database. If *Klebsiella* sp. genomes were found to be <95% homologous, then they are not assigned an ST, and the platform determine whether the species correspond to any of the other species included in the ANI analysis. In addition, if MLST for *K. variicola* is implemented in laboratories interested in determining STs among *K. variicola* isolates, the isolates negative for *pyrG* by PCR amplification suggest the possibility that these isolates do might not correspond to *K. variicola*.

**Figure 5:**
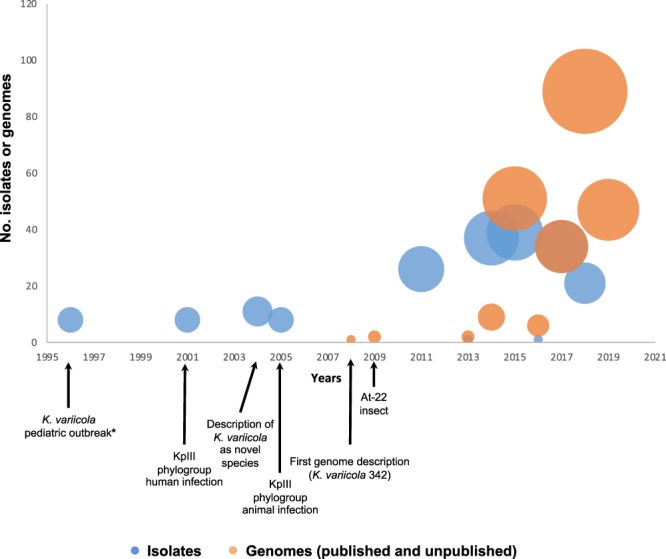
Timeline description of *K. variicola* isolates and genomes described in public databases. The KpIII group corresponds to *K. variicola*. The *K. variicola* pediatric outbreak marked with asterisk corresponds to the isolation date of the clinical isolates.

Figure 5 depicts the epidemiological history of *K. variicola* based on the year of publication. Although the first three works related to *K. variicola* were published in 2001, 2004 and 2008, the bacteria identified were isolated in the previous years. An outbreak of *K. variicola* was described in 2007, but the isolates were obtained in 1996^33^. These isolates are related to bloodstream infections from a pediatric outbreak in Mexico. The first *K. variicola* isolate (801) was obtained on April 09 of 1996 (Fig. 5). Subsequently, this isolate was subjected to WGS and used to develop a PCR-multiplex assay^12^ and considered as ST1 in this work. To describe *K. variicola* as a new bacterial species in 2004^1^, a phylogenetic analysis that included *K. pneumoniae* isolates identified three phylogroups, named KpI, KpII and KpIII, was described in 2001^44^. The KpIII phylogroup was mentioned by Rosenblueth *et al*. (2004), which corresponds to *K. variicola*^1^. In the next year (2005), several isolates belonging to the KpIII group and obtained from veterinary infections (dog, monkey and bird) in the Netherlands also correspond to *K. variicola*^45^. The first *K. variicola* misclassification corresponded to *K. pneumoniae* 342, and several reports subsequently described this isolate as *K. variicola*. One year later, *K. variicola* At-22 was obtained from leaf-cutter ant-fungus gardens in South America^31^, and numerous *K. variicola* genomes have since been described (Fig. 5). Several studies have considered the misclassification existing within the *Klebsiella* genus^12,13,15,16^ it has allowed that genomes that correspond to *K. variicola* could be correctly identified and updated. The population structure, virulence and antibiotic resistance of *K. variicola* has been addressed through WGS^34^, and the BioProject (PRJEB10018) recently identified *K. variicola* clinical isolates in several European countries (Fig. 1). In general, rigorous molecular epidemiological studies of *K. variicola* require an MLST scheme, which allow for surveillance of multidrug-resistant and hypervirulent clones.

### Comparison of MLST schemes

The *K. pneumoniae* MLST scheme works for *K. variicola* and different studies have applied the *K. pneumoniae* MLST scheme for typing *K. variicola* isolates^13,14,18^. However, for an unknown reason, a large number of *K. variicola* isolates were not assigned an ST, considering that the genomes have been determined^13,18^. In this study, both MLST schemes were compared using *K. variicola* genomes.

*K. pneumoniae* MLST was developed in 2005, when new species closely related to *K. pneumoniae* were still unknown. Of the seven locus (*rpoB*, *gapA*, *mdh*, *pgi*, *phoE*, *infB* and *tonB*) of the *K. pneumoniae* MLST scheme, three (*rpoB*, *pgi* and *phoE*) are shared with the *K. variicola* MLST scheme. The *tonB* was not considered for *K. variicola* MLST because in the case of *K. pneumoniae*, several nucleotides have been inserted at this locus. However, the *K. pneumoniae* MLST scheme eliminates inserted nucleotides (https://bigsdb.pasteur.fr/klebsiella/klebsiella.html). Such nucleotide insertion was also identified in the *tonB* gene of *K. variicola*. Considering its role in the binding and transport of siderophores, *tonB* might be undergoing selection pressure. These proteins could be directly involved in pathogenicity^46^, which has been observed an increase in pathogenicity for *Klebsiella* spp., with strains of *K. pneumoniae*, *K. quasipneumoniae* and *K. variicola* being hypervirulent^35,47–49^. Further investigation is needed to this respect.

In the case of *rpoB, leuS* and *fusA* and *pyrG* genes were considered in the *K. variicola* MLST scheme, because the first three genes were proposed for phylogenetic differentiation of *K. pneumoniae*, *K. variicola* and *K. quasipneumoniae*^2^. In the case of *pyrG*, a GTP synthase class-I is absent in *K. pneumoniae* and as mentioned above, this gene may contribute to the proper identification of *K. variicola*.

Moreover, 178 of the *K. variicola* genomes included in this study were subjected to ST assignments in the *K. pneumoniae* MLST scheme, of them 11.8% of the genomes were not assigned to an ST because they corresponded to new ones. *K. variicola* genomes with an assigned ST were analyzed through goeBURST analysis using the *K. pneumoniae* MLST database. This result showed a clear dispersion of *K. variicola* isolates, considering that the STs assigned to *K. variicola* isolates are shared ST profile with the *K. pneumoniae* isolate deposited in the *K. pneumoniae* MLST database (Fig. S1). However, the goeBURST analysis using the *K. variicola* MLST database revealed that some of these *K. variicola* isolates are close related to each other when they were separated by an SLV. These data emphasize that it is difficult to establish genetic relationships among *K. variicola* isolates using the *K. pneumoniae* MLST scheme because *K. variicola* isolates are related to those of *K. pneumoniae* instead of those of their own species. In summary, using the same MLST scheme for closely related bacterial species is not discriminative.

Finally, the allelic profile of *K. variicola* genomes was analyzed using both *K. pneumoniae* and *K. variicola* MLST schemes (Fig. S2). A heatmap shows higher variability when the *K. variicola* MLST scheme was applied in the examined *K. variicola* genomes.

### Impact of *K. variicola* in different environments

In the last year, *K. variicola* has been strongly suggested to cause serious infections in humans, including hospital outbreaks, which has increased the phenotypic identification of ESBL- and carbapenemase-producing in environmental and clinical isolates. An increase in virulence has also been described, including the identification of hypermucoviscous^50^ and hypervirulent^33^ strains and those causing high mortality in a pediatric outbreak^51^. Additionally, colistin-resistant isolates have been reported, and the chromosomal mechanisms that are responsible for this phenotype have been identified^35^. Outside the clinical environment, signs of infection in farm and wild animals have been observed and strongly correlated with some insects. *K. variicola* is widely distributed among different groups of plants, mainly those consumed by humans, which facilitates the transfer of these bacteria from plants to humans. Accordingly, *K. variicola* species has been proposed to constitute a cross-kingdom bacterium. In the environment, *K. variicola* has been detected in rivers and on inert surfaces, and a review of *K. variicola* details the wide range of environments in which *K. variicola* has been detected as well as its use in industry^6^. In addition to the nitrogen-fixation capacity of *K. variicola*, these findings reveal clear differences from other species of the *Klebsiella* genus, mainly *K. pneumoniae*^8,16,17,29^.

## Conclusions

*K. variicola* is a bacterial species that has been misclassified as *K. pneumoniae* for years, and it has also been misclassified as *K. quasipneumoniae*. *K. variicola* and *K. pneumoniae* share clinical settings and both are endophytes in plants and cause infections in livestock and wild animals. In clinical settings, *K. variicola* shows clear differences with regard to infection, highlighting the importance of early diagnosis. There are several approaches for the proper identification of *K. variicola* among *K. pneumoniae* complex. The database of *K. variicola* MLST will allow the molecular epidemiology of this bacterial species and establish the identification of possible pandemic clones. In addition, this study reveals a possible route of transmission of this bacterial endophyte from plants to humans. Although this phenomenon was previously identified, several results of the present study strengthen this evidence. *K. variicola* and *K. pneumoniae* are closely related bacterial species which must be stored separately, preventing one from masking the other and the relationships among the same bacterial species can being found.

## Materials and Methods

### *K. variicola* MLST scheme

For the development of the *K. variicola* MLST scheme, seven housekeeping genes were selected after AMPHORA (AutoMated PHylogenOmic infeRence) analysis^51^. The analysis was performed using thirty-three *K. variicola* proteomes. Primer pairs were successfully designed (using the Primer-BLAST tool https://www.ncbi.nlm.nih.gov/tools/primer-blast/) for PCR amplification and sequencing of an internal position of the seven genes.

### *K. variicola* genomes

In total, 226 *K. variicola* genomes were obtained from public GenBank/ENA databases (01/05/2019). These genomes were validated as belonging to *K. variicola* using the Average Nucleotide Identity (ANI) tool^45^. The reference genomes for each bacterial species included are *K. pneumoniae* MGH78578 (GenBank Accession number CP000647.1), *K. quasipneumoniae* 18A069 (GenBank Accession number CBZM000000000), *K. variicola* At22 (GenBank Accession number CP001891.1) and *K. quasivariicola* KPN1705 (GenBank Accession number CP022823.1).

### Sequence type determination in *K. variicola* genomes and isolates

*K. variicola* MLST scheme STs were determined for 226 *K. variicola* genomes and a collection of 28 *K. variicola* isolates obtained from plants and humans in Mexico. In these isolates, the PCR amplification products were carried out following the instructions described in this study (Table 1). The nucleotide sequences of the 7 MLST genes were obtained using BigDye^TM^ Terminator v3.1 and analyzed with the Applied Biosystems 3130 platform. For more details of the PCR conditions visit the page of *K. variicola* MLST scheme (http://mlstkv.insp.mx).

### goeBURST analysis

The goeBURST-1.2.1^52^ program was used to analyze STs of *K. variicola* isolates and to assign isolates to a clonal complex (CC). A clonal complex is defined as a set of similar STs with six identical locus. A CC is formed by the founder ST and its SLVs.

### Phylogenetic analysis

The phylogenetic analysis was performed using the 7-locus *K. variicola* MLST concatenated genes, and a Maximum Likelihood phylogeny tree was generated using Mega software v7.0.26. The Tamura-Nei model with discrete Gamma distribution was applied to model evolutionary rate differences among sites (4 categories (+G, parameter = 0.1133))^53^.

### Implementation of ANI by proper *K. variicola* bacterial species

ANI tool^45^ analysis was implemented in the *K. variicola* MLST homepage (http://mlstkv.insp.mx) to ensure the correct identification of *K. pneumoniae*, *K. quasipneumoniae*, *K. variicola* and *K. quasivariicola*. The reference genomes for each bacterial species analyzed using ANI are *K. pneumoniae* MGH78578 (GenBank Accession number CP000647.1), *K. quasipneumoniae* 18A069 (GenBank Accession number CBZM000000000), *K. variicola* At22 (GenBank Accession number CP001891.1) and *K. quasivariicola* KPN1705 (GenBank Accession number CP022823.1).

### Molecular epidemiology of *K. variicola*

The molecular epidemiology of susceptible, ESBL- and carbapenemase-producing *K. variicola* was established according to respective publications (Supplementary Dataset). In addition, isolates with genomic data but unpublished β-lactamases were determined using ResFinder based on acquired antimicrobial resistance genes (https://cge.cbs.dtu.dk/services/ResFinder/)^54^.

### Comparison of MLST typing schemes

The MLST *K. pneumoniae* scheme was used to determine STs for 178 *K. variicola* genomes included in the study. goeBURST analysis was carried out using the allelic profile. A heatmap was drawn to determine the distances between the allelic profiles of the seven genes of the *K. pneumoniae* and *K. variicola* MLST schemes and Multiple Experiment Viewer MeV version 4.8.1 software.

## Supplementary information

Fig. S1

Fig. S2

Supplementary Dataset
