# Supplementary material for: Molecular epidemiology of Klebsiella variicola obtained from different sources
Source: Sci Rep. 2019 Jul 23;9:10610. doi: 10.1038/s41598-019-46998-9 (PMC6650414; doi:10.1038/s41598-019-46998-9)
Supplement: Supplementary file 2 — Fig. S2 [file 41598_2019_46998_MOESM2_ESM.pdf]

Molecular epidemiology of *Klebsiella variicola* obtained from different sources

Barrios-Camacho Humberto[1&], Aguilar-Vera Alejandro[2&], Beltran-Rojel Marilu[1], Aguilar-Vera Edgar[3], Duran-Bedolla Josefina[1], Rodriguez-Medina Nadia[1], Lozano-Aguirre Luis [4], Perez-Carrascal Olga Maria[4], Rojas Jesús[3], and Garza-Ramos Ulises[1*]

Corresponding author

Garza-Ramos Ulises (ulises.garza@insp.mx) Instituto Nacional de Salud Pública (INSP), Centro de Investigación Sobre Enfermedades Infecciosas (CISEI), Laboratorio de Resistencia Bacteriana, Cuernavaca, Morelos, México.

[1]Instituto Nacional de Salud Pública (INSP), Centro de Investigación Sobre Enfermedades Infecciosas (CISEI), Laboratorio de Resistencia Bacteriana, Cuernavaca, Morelos, México.

[2]Universidad Nacional Autónoma de México, Centro de Ciencias Genómicas, Programa de Genómica Funcional de Procariotes, Cuernavaca, Morelos, México.

[3]Instituto Nacional de Salud Pública (INSP), Centro de Información para Decisiones en Salud Pública (CENIDSP).

[4]Universidad Nacional Autónoma de México, Centro de Ciencias Genómicas, Programa de Genómica Evolutiva, Cuernavaca, Morelos, México.

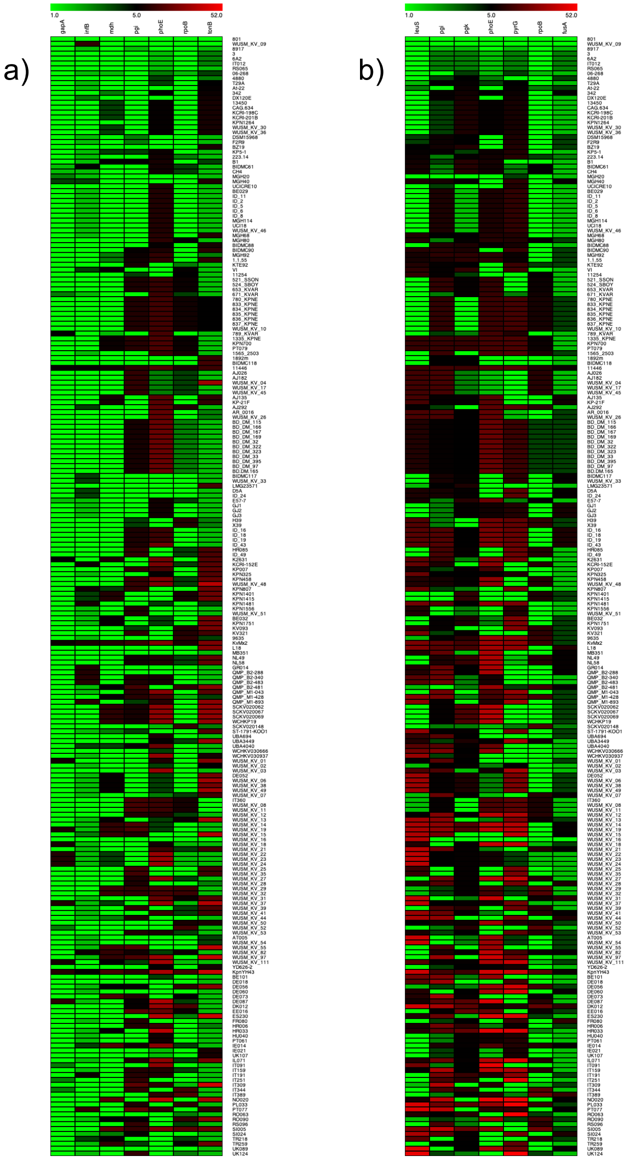

Fig. S2. Heatmap showing the allelic profile variation of *K. variicola*. a) Allelic profile variation of *K. variicola* genomes using the *K. pneumoniae* MLST scheme. b) Allelic profile variation of *K. variicola* genomes using the *K. variicola* MLST scheme.
